# Appeal: the protection of ancient tree species around the world, taking qilian juniper (*Juniperus przewalskii*) as an example

**DOI:** 10.1016/j.heliyon.2022.e10232

**Published:** 2022-08-14

**Authors:** Wenqin Zhao, Heng Yang, Jieshi Tang

**Affiliations:** a College of Life Sciences, Shihezi University, Shihezi, China; b Xinjiang Production and Construction Corps Key Laboratory of Oasis Town and Mountain-basin System Ecology, Shihezi, China; c College of Life Science, Sichuan University, Chengdu, China

**Keywords:** *Juniperus przewalskii*, Tree growth, Plant reproduction, Heredity, Physiology, Phytochemistry, Conservation

## Abstract

*Juniperus przewalskii* (the Qilian juniper) is a dominant species in the northeast region of the Qinghai-Tibet Plateau. As such, it is of great significance for maintaining the balance and long-term stability of the ecosystem in this biodiversity hotspot. In this paper, we review the literature related to *J. przewalskii* published in the China National Knowledge Infrastructure and Web of Science. Here, we summarize the main research achievements with regard to this species from ten aspects: tree morphology and phenology, population structure and life history, radial growth and climate response, tree-ring chronology-based history reconstruction, soil physical and chemical properties, chemical composition and activity, physiological ecology, biogeography, breeding and propagation techniques, and occurrence and control of diseases and pests. Considering the ecological importance and research value of *J. przewalskii*, as well as the shrinking population size, we discuss future research prospects and directions for the conservation of *J. przewalskii* from four aspects: global climate change, human disturbance, tree regeneration, and pest control. This work provides an important reference for conservation studies of alpine tree species in other biodiversity hotspots around the world.

## 1 Introduction

The Qilian juniper (*Juniperus przewalskii* Kom.) is a tree species endemic to the Qinghai-Tibet Plateau, which is one of the global biodiversity hotspots. This species is mainly distributed in the Songpan area of northern Sichuan, the southern region of the Hexi Corridor in Gansu, and the eastern, northern, and northeastern regions of Qinghai (Liu and Yang, 2015). *Juniperus przewalskii* generally grows on semi-sunny or sunny slopes at different elevations (approximately 2500–4000 m), and the lifespan of individuals can exceed 3,000 years (Yang et al., 2014, 2017; Liu et al., 2019; Liu et al., 2022). As a Quaternary relict plant and dominant species, and because of its cold and drought resistance, *Juniperus przewalskii* plays a key role in the ecological maintenance of the complex mountain ecosystem in the Qilian Mountains of the Qinghai-Tibet Plateau (Chen et al., 2006; Zhang et al., 2009; Zhuang et al., 2019; Fang et al., 2021). In addition, the essential oil obtained from Qilian juniper is widely used as medicine, spice, and food, and has important uses in agriculture and forestry (Huang et al., 2004; Watanabe et al., 2011). Due to its hemostatic and antitussive effects, the oil is used in Chinese and Tibetan medicine, and can be used to treat nephritis, arthritis, and uterine bleeding (Liu and Li, 2013).

In recent years, the effects of climate change and anthropogenic activities (such as tree felling and grazing activities) have led to difficulties in seedling regeneration and the spread of diseases and pests. As a result, the natural population of *J. przewalskii* has been seriously threatened, and there is an urgent need to protect this plant resource (Dong et al., 2020; Li, 2020, Li, 2020; Lu, 2021). This review summarizes the findings of representative studies on *J. przewalskii* populations published in the China National Knowledge Infrastructure and Web of Science. This review can help our domestic and foreign peers understand the research status and challenges of this plant group. We also discuss important scientific issues that are worth exploring in order to provide scientific reference for the protection and sustainable utilization of this plant resource.

## 2 Morphological and phenological studies

Qilian juniper belongs to the genus *Juniperus* in the family Cupressaceae. The trees can grow to a height of up to 12 m and form sparse shrub foliage. The trunk is straight or slightly twisted, and splits into strips that fall off. The branchlets do not droop, and are quadrangular or square-shaped. The leaves can be spiny or scaly; young trees have spiny leaves, whereas big or old trees primarily have scaly leaves. The spiny leaves have three alternating whorls and are triangular-lanceolate, concave on the top and rounded below, or with ridges on the top. The scaly leaves show the opposite physical characteristics. The trees are monoecious with oval male corpuscles, yellow pollen, ovoid or nearly spherical cones, and seeds that are blunt on both ends (Flora of China, 1974). The flowering period of *J. przewalskii* occurs relatively late (June–July) and last for a relatively long time (15–25 days), and the trees begin to bear fruit at the age of approximately 15 years. The cone develop within two years, and the fruit quantity increases with tree age. The fruit yield tends to stabilize when trees are approximately 50 years old, and the continuous fruiting pattern changes to an alternating pattern, with alternating big and small years (Gao and Ling, 2020). An accurate understanding of the morphological and phenological characteristics of *J. przewalskii* can provide a taxonomic foundation for species identification and resource conservation.

## 3 Population structure and life history

Population structure is an important component of plant population ecology, as it reflects the spatial changes in plant populations and the trends of population dynamics and community succession. Liu and Yang (2015) studied the life history of *J. przewalskii* populations in the Lenglongling Danjun horse farm forest area using life table and survival analysis. The results showed that the *J. przewalskii* population was in decline; the population survival rate and mortality density function were in monotonic decline, and the cumulative mortality and risk function showed a monotonous increase with an increase in size class. Tian et al. (2015) analyzed the diameter at breast height within the class structure of a *J. przewalskii* population on the north slopes of the Qilian Mountains. The results showed that young trees and seedlings were scarce, and that adult individuals accounted for a large proportion of the population. In general, the population structure was an inverted pyramid. This indicated a declining trend and highlighted the need for the protection of this species. Zhan (2015) investigated the spatial distribution of a *J. przewalskii* population on the northern slopes of the Qilian Mountains and found an aggregated distribution in high-altitude areas, along with random distribution in low-altitude areas. This may be because at high altitudes, an aggregated distribution is beneficial for *J. przewalskii* to resist the extreme environment, consolidate the nutrient requirements, and enhance population competitiveness. In contrast, frequent grazing activities and more disturbance from humans and livestock can destroy the habitat conditions required for seed germination and seedling growth. The spatial structure of random distribution was a result of this self-thinning effect. In addition, climate change is also responsible for this distribution pattern, as lower elevations are more sensitive to the effects of climate change.

## 4 Radial growth and climate response

There are controversies regarding the climatic factors that limit the radial growth of trees on the alpine timberline zone in the northeast region of the Qinghai-Tibet Plateau. Qin et al. (2013) developed four equivalent tree-ring width chronologies and showed that there was no significant difference in the growth of *J. przewalskii* during 1956–2011. The authors also developed a long-term growth model for this species from the 1110s to the 2010s. The main effect of climate on the radial growth of *J. przewalskii* was through precipitation variability. The analysis of missing rings revealed that the radial growth was more sensitive to climate change in trees at the top of the forest line than in those at other elevations, and that poor radial growth was closely related to severe drought events. Peng et al. (2010) used the correlation analysis between standard chronology and climatic factors to reveal the various effects of climate on the radial growth of *J. przewalskii* in the east-west direction of the Animaqing Mountains. The results of response function analysis showed that there were regional differences in tree growth and climatic factors at different sampling sites across the study area. Temperature and precipitation were important factors affecting tree growth in the western (dry) upper forest line. Tree growth along the upper tree line was greatly limited by temperature in the central region, and by precipitation in the eastern region (wet). He et al. (2016) suggested that in the northeastern Qinghai-Tibet Plateau, the ring width of *J. przewalskii* was mostly controlled by moisture, and was not affected by altitude. The radial growth period of *J. przewalskii* is in June and July, and the differentiation period of all xylem cells ends in mid-August (He et al., 2013; Gao and Ling, 2020). As such, precipitation and relative humidity are the main limiting factors for tree radial growth in the northeastern Qinghai-Tibet Plateau (Yang et al., 2014).

## 5 Historical climate reconstruction based on tree rings

Studying the history of regional drought can help us understand the hydroclimatic changes being caused by current global warming and predict future hydroclimatic shifts. Gou et al. (2015) reconstructed the self-calibrating Palmer Drought Severity Index (scPDSI) from May to July based on two tree-ring width chronologies of *J. przewalskii* for nearly 1000 years. The results showed that the Little Ice Age (LIA) was a relatively dry period from the 15th to the 18th centuries, and that the last two centuries were a relatively wet period. This reconstruction was consistent with other tree-ring-based hydroclimatic reconstructions in the northeastern Tibetan Plateau and the corresponding century-scale solar activity during the LIA. The three extreme drought periods recorded by tree rings (AD 1260s–1340s, 1430s–1540s, and 1640s–1740s) also correspond to the minimum periods of three solar activities in Wolf, Spörer, and Maunder. The results of multi-tape and wavelet analyses further confirmed the relationship between regional hydroclimatic variability and solar activity stress. Tian et al. (2007) reconstructed the drought history (AD, 1855s–2001s) of the Qilian Mountains in northwest China by calibrating with a linear interpolation through four PDSI grid values nearest to the sampling site. Their reconstruction extended the drought history of this area and revealed that the most severe drought occurred in the 1920s. In the context of the drought history of western China, the extreme drought during 1925–1931 is consistent over a large region surrounding Northwestern China. Yang et al. (2021) reported that superimposed on a persistent drying trend since the mid-Holocene, a rapid decrease in moisture availability between ∼2000 and ∼1500 BCE caused a dry hydroclimatic regime from ∼1675 to ∼1185 BCE. The mean precipitation during this period has been estimated at 42 ± 4% and 5 ± 2% lower than that during the mid-Holocene and the instrumental period, respectively.

## 6 Soil physical and chemical properties

The Qilian juniper plays a very prominent role in the water retention and soil conservation of mountain ecosystems in cold and arid regions (Li, 2020). Zhang et al. (2013) used a combination of field sampling and indoor analysis to study the physical and chemical properties of soil and changes in the organic carbon content and nutrient compositions of different woodlands in the Binggou Valley in the upper reaches of Heihe. The results showed that soil bulk density, proportion of physical sand, CaCO_3_ levels, and soil pH increased with soil depth, whereas total nitrogen, total phosphorus, organic carbon, soil aggregates, and cation exchange capacity decreased. The changes in the aforementioned indicators (from small to large) in different woodlands were in the following order: *Picea crassifolia* < alpine shrub < *J. przewalskii*. In Gao et al. (2016) tested the soil physical and chemical properties of five vegetation types in the Tianlaochi watershed upstream of the Heihe River. The results across vegetation types were as follows: soil bulk density: grassland (steppe, 1.12 g/cm^3^; subalpine meadow, 1.00 g/cm^3^) > shrub land (subalpine shrub, 0.76 g/cm^3^) > forest land (*J. przewalskii*, 0.75 g/cm^3^; *P. crassifolia*, 0.50 g/cm^3^); total porosity of soil: forest land (*P. crassifolia*, 81.29%; *J. przewalskii*, 76.61 %) > shrub land (subalpine shrub, 72.63 %) > grassland (subalpine meadow, 62.28%; steppe, 57.65%); soil organic matter content: *P. crassifolia* (136.87 g/kg) > subalpine shrub (119.96 g/kg) > subalpine meadow (94.67 g/kg) > *J. przewalskii* (70.69 g/kg) > steppe (43.94 g/kg). The soil organic matter content was generally higher on the shady slope than on the sunny slope. These results indicate that *J. przewalskii* plays an important role in this mountain ecosystem.

## 7 Chemical constituents and biological activities

The plant tissues of Cupressaceae are rich in essential oils. Li et al. (2021) analyzed the essential oil composition of *J. przewalskii* using gas chromatography-mass spectrometry. The same study also evaluated the antibacterial activity of these essential oils by filter paper fumigation, the minimal inhibitory concentration (MIC) test, and the minimum bactericidal/fungicidal concentration (MBC/MFC) test. A total of 47 chemical constituents were identified from the essential oils, These included sabinene (19.59%), α-cadinol (11.81%) (-)-4-terpinol (10.90%), γ-terpinene (5.55%), citronellol (5.06%), myrcene (3.705%), terpinolene (3.58%), and d-limonene (3.34%), among others. The bacteriostatic activity test with bacteria showed that the MIC and MBC of *Staphylococcus aureus* and *Bacillus subtilis* were 2.34 μL/mL and 4.68 μL/mL, respectively. The MIC of *Alcaligenes faecalis* and *Escherichia coli* were both 4.68 μL/mL, and the MBC of *A. faecalis* and *E. coli* were 4.68 μL/mL and 9.36 μL/mL, respectively. Among fungi, the MIC and MFC of *Penicillium expansum* and *Fusarium sulphureum* were 0.875 μL/mL and 1 μL/mL, respectively, and the MIC of *Alternaria alternata* and *Trichosporon roseum* were 1.125 μL/mL and 1.25 μL/mL, respectively. Wang et al. (2010) also isolated two new diterpenoids and 17 known terpenoids from *J. przewalskii*, and identified their structures by infrared spectroscopy, mass spectroscopy, H-1, C-13 nuclear magnetic resonance (NMR) spectroscopy, and two-dimensional NMR spectroscopy. Among the compounds isolated, 3 alpha-hinokiol and 3 alpha-hydroxymannol have strong anti-tumor activity against cervical cancer (HeLa) and human ovarian cancer (HO-8910) cell lines, respectively. These results provide a scientific basis for the development of microbial inhibitors from *J. przewalskii* extracts for the treatment of human tumors and other related diseases.

## 8 Physiological and ecological research

The Qilian juniper primarily grows in high-altitude areas. During long-term evolution in alpine habitats, alpine plants typically develop specific physiological adaptations to resist cold and drought stress. Chen (2008) found that with increasing altitude, the levels of soluble sugar (SS), soluble protein (SP), and malondialdehyde (MDA), as well as the activities of peroxidase (POD), superoxide dismutase (SOD), and catalase (CAT) increased in the leaves of *J. przewalskii* plants. Moreover, the physiological characteristics of *J. przewalskii* that help it adapt to the extreme alpine environment—such as the cuticular thickness of the leaf epidermis, palisade cell coefficient, stomatal density, and intercellular area—also increased with altitude. Chen (2006) demonstrated that nitric oxide (NO) acts as a signal molecule to activate the antioxidant system and induce frost resistance in *J. przewalskii*. The frost resistance of leaves is closely associated with the levels and activities of antioxidant enzymes. The antioxidant system in leaves plays an important role in limiting the production of free radicals and protecting the integrity of cell membranes. The cold tolerance of *J. przewalskii* is related to the enhanced ability of its antioxidant system to scavenge or detoxify reactive oxygen species (ROS). The accumulated ROS are a component of cold acclimation and may induce the antioxidant defense system, thereby allowing the leaves to gain cold tolerance. Su (2012) measured the photosynthetic capacity of *J. przewalskii* leaves in Tulugou National Forest Park and discovered two patterns of diurnal variation in the photosynthetic rate: single peak and double peak. The unimodal (single peak) pattern occurred in April, whereas the bimodal (double peak) pattern occurred in the other months. The results of path analysis and correlation analysis showed that stomatal conductance and temperature were the main physiological and ecological factors that determined the photosynthetic rate of *J. przewalskii*.

## 9 Biogeographic studies

Phylogeographic studies can elucidate the relationship between species evolution and climatic history and geological events. These analyses can help us identify species refuges during Quaternary glacial periods and their expansion routes during interglacial periods (Avise et al., 1987). Zhang et al. (2005) studied the phylogeography of *J. przewalskii* based on chloroplast DNA *trn*T-*trn*F sequences. The results showed that there were several refuges of *J. przewalskii* during the Quaternary ice age, and that the current pattern of genetic diversity across populations is mainly due to founder effects and species bottlenecks. Through combined analysis with *trn*S-*trn*G sequences, six cpDNA haplotypes were identified, and the phylogenetic network was constructed. The evolutionary relationships indicated that the most widely distributed haplotype (haplotype A) is not the ancestor of the other haplotypes. There were several haplotypes of *J. przewalskii* at the edge of the Qinghai-Tibet Plateau, which indicated that this area was the glacial refuge of *J. przewalskii*. In contrast, the haplotypes on the Qinghai-Tibet Plateau platform were almost identical, which provided support for the post-glacial re-colonization of this area by *J. przewalskii* populations. Population expansions at the edge of the interglacial plateau and their discontinuous distribution following anthropogenic disturbance in the Holocene were the main reasons for the current spatial distribution of *J. przewalskii* cpDNA haplotypes (Zhang et al., 2010). Li et al. (2011) used 8 nuclear loci to study the demography of *J. przewalskii* populations. The results indicated that although the species seemed to have experienced post-glacial expansion during the Quaternary period, the population size did not change significantly during the Pleistocene.

## 10 Softwood cuttings and propagation techniques

*J. przewalskii* exhibits resistance to drought and cold. Due to the continued afforestation efforts in the Qinghai-Tibet Plateau, there is an increasing demand for *J. przewalskii* seedlings (Xu, 2007; Zhang, 2021). At present, *J. przewalskii* is propagated mainly through two methods: softwood cuttings and seed germination. Li (2021) performed germination experiments on *J. przewalskii* seeds (tree age, 70–120 years) using various methods, including freezing, variable temperature stratification, mixed sand stacking, chemical treatment, and seed soaking combined with snow storage. Seed soaking combined with snow storage resulted in the highest seedling emergence rate (approximately 44%). The freezing and variable temperature stratification methods also showed significant success. Chen (2021) investigated propagation through softwood cuttings using semi-lignified cuttings collected from *J. przewalskii* plants (age, 6–10). The inner branches had a length of 10–30 cm and a thickness of 0.5–0.8 m. Before cutting, the base of the cuttings was soaked in root powder solution (50–100 mg/kg) for 12–24 h, following which the cuttings were immersed in carbendazim or potassium permanganate solution (2–4‰) for 5 min for disinfection. More than 80% of the cuttings survived these treatments. These measures significantly improved the success rate of *J. przewalskii* breeding and greatly reduced the time and economic cost compared to previous methods.

## 11 Diseases and pests and their control

In addition to climate change, diseases and insect pests also seriously affect the survival and growth of *J. przewalskii*. Dong et al. (2020) reported that *J. przewalskii* can be infected by 17 pathogens, including two pathogens that parasitize roots (*Fusarium oxysporum* and *Rhizoctonia solani*), 12 that are parasitic on tree trunks, wood, and fallen trees (*Tubifera microsperma*, *Badhamia utricularis*, *Stemonitis splendens*, *Arcyria incarnata*, *Craterium leucocephalum*, *Fuligo septica*, *Ischnoderma resinosum*, *Heterobasidion annosum*, *Physarum globuliferum*, *Physarum rigidum*, *Pleurotus nidulans*, *Pholiota spumosa*), 2 that are parasitic on branches (*Gymnosporangium yamadai* and *Usnea diffracta*), and 1 that is parasitic on leaves (*Gymnosporangium clavariiforme*). The diseases caused by these parasites include seedling blight, branch disease, xylem rot, pear rust, apple rust, and leaf blight. The main pests of *J. przewalskii* include *Megastigmus sabinae* Xu et He, *Semanotus bifasciatus*, *Cinara sabinae*, *Parocneria sabinae*, *Larva Holotrichiae* and *Agrotis ipsilon* (Li, 2020; Lu, 2021). When planted in a nursery, the seedlings should be sprayed with the same volume of Bordeaux mixture (1:1:1) once every 7 days within 5–7 days after they are unearthed. Spraying a total of 2–3 times can prevent the occurrence of pests and diseases. If blight occurs at this time, the plants can be sprayed with thiophanate-methyl solution (70%) once every 6 days to control the infection. Three drugs (thiophanate-methyl, mancozeb, and carbendazim) can be sprayed alternately if stem and leaf rot are found during the peak growing season. When pests occur, they can be captured manually. For underground insect pests, medicines can injected into the seed bed of the injured seedlings. The wound can be sprayed with EC (3911) solution, which can quickly eliminate pests such as *Agrotis ipsilon* (Wang, 2018). Although chemical and artificial control can effectively prevent the occurrence of diseases and infection by insect pests in *J. przewalskii*, the high economic costs and problems associated with chemical pollution are not conducive to sustainable development. Therefore, active biological control would be a better solution in the future.

## 12 Outlook

In recent years, some progress has been made in the research on *J. przewalskii*. However, the protection efforts for this species need to be strengthened further. Future conservation research should proceed in following directions: (Ⅰ) landscape genomic studies on adaptive genetic variation in the species can provide a theoretical basis (Bay et al., 2018) for the development of adequate conservation measures in the face of global warming (such as assisted gene flow and/or assisted migration of *J. przewalskii*); (Ⅱ) with increase in interference from human activities, appropriate laws and regulations or protected areas should be established to prevent human and livestock from disturbing and destroying natural habitats (Tang et al., 2022); (Ⅲ) mutualist fungal partners of *J. przewalskii* (such as arbuscular mycorrhizal fungi) should be actively investigated in order to promote seedling growth and population expansion (Shi et al., 2020); (Ⅳ) the development of environment-friendly biological pesticides will allow the harmonious development of humans and nature (Shao et al., 2018). Strengthening our research efforts on the protection of *J. przewalskii* in the Qinghai-Tibet Plateau will allow the sustainable utilization and development of this plant resource and help maintain ecosystem stability in this biodiversity hotspot.

## Declarations

### Author contribution statement

All authors listed have significantly contributed to the development and the writing of this article.

### Funding statement

Wenqin Zhao was supported by the National Natural Science Foundation of China [32060374], the Talent Initiation Project of Shihezi University [RCZK201952].

### Data availability statement

Data will be made available on request.

### Declaration of interest's statement

The authors declare no conflict of interest.

### Additional information

No additional information is available for this paper.
